# Advances in Postharvest Preservation and Quality of Fruits and Vegetables

**DOI:** 10.3390/foods12091830

**Published:** 2023-04-28

**Authors:** Juan Luis Valenzuela

**Affiliations:** Research Centres CIAIMBITAL and CeiA3, Department of Biology & Geology, Higher Engineering School, University of Almería, 04120 Almería, Spain; jvalenzu@ual.es

Numerous agricultural regions face the daunting task of providing high-quality fresh fruits and vegetables to increasingly competitive markets. In addition, they must contribute to sustainable production by minimizing postharvest losses and extending the shelf life of their produce. To tackle this challenge, a postharvest physiology and technology approach is required, which provides the necessary knowledge to introduce innovative technologies and solutions that aid in preserving the quality of fruit and vegetable products, while minimizing losses and wastage. As a result, there has been a growing interest in applying the latest technologies and deepening the understanding of postharvest physiology, with the belief that only a scientific approach can provide the necessary solutions to meet the demands of a highly competitive and exacting world. The end goal of postharvest physiology and technologies is to ensure that fruits and vegetables attain new markets while maintaining their quality and minimizing postharvest losses. This goal is attainable, provided that there is an effective transfer of knowledge to the production sector.

Post-harvest is a crucial part of agricultural activity aimed at maximizing the quality and shelf life of agricultural products in general, and fruits and vegetables in particular, ensuring that they reach the market in the best possible condition. Proper post-harvest management is essential for the success of the marketing of these products, as it directly affects their quality. If the products are not handled correctly, they can lose quality, taste, flavour, texture, and nutrients, resulting in economic losses for the producers and, obviously, lower-quality products for consumers.

There is a close relationship between postharvest management and the loss and waste of fruit and vegetables [1]. Postharvest losses occur when the quality and quantity of harvested crops are reduced or lost before they reach the consumer. This is mainly due to poor handling and storage practices, inadequate transport, and lack of infrastructure [2]. Food waste, on the other hand, occurs when food is discarded or not used by consumers, retailers, or other stakeholders in the food supply chain [2]. Although post-harvest losses occur earlier in the supply chain, they can contribute to food waste if retailers or consumers discard sub-standard products [3].

Proper post-harvest management can help reduce both post-harvest losses and food waste. By improving handling and storage practices, farmers can help prevent crop damage that can lead to spoilage and losses. Efficient transport and infrastructure are essential to ensure that horticultural produce arrives at its destination in satisfactory quality, health, and undamaged condition.

Overall, effective post-harvest management is essential to reduce both post-harvest losses and food waste. By improving the quality and quantity of harvested crops, we can help get more food to those who need it, while reducing waste and protecting the environment.

New technologies and innovation play an essential role in preserving the quality of fruits and vegetables after harvest. Innovations can help extend the shelf life of fruits and vegetables, reduce spoilage, and maintain quality and nutritional value. Many diverse examples of new technologies that have been developed to preserve the quality of fruits and vegetables is the use of nanotechnology. Nanoparticles, such as silver and zinc oxide, have antimicrobial properties that can help reduce the growth of bacteria and fungi on the surface of fruits and vegetables, thus reducing spoilage and extending their shelf life [4]. Another example is the use of smart packaging, which is designed to monitor and regulate the environment around the product, such as temperature and humidity, to ensure optimal conditions for preserving the quality of fruits and vegetables [5].

The content of this Special Issue offers a varied and interdisciplinary perspective on the topic of postharvest quality and preservation of fruits and vegetables. It encompasses research that is specifically focused on studies aimed at maintaining the quality and prolonging the life of fruit and vegetable products. Thus, there are different articles ranging from technologies based on physical methods to technologies based on the application of different compounds.

Guo et al. [6] provide a comprehensive overview of the potential of essential oils and plant extracts as natural preservatives to extend the shelf life of mushrooms. The authors discuss the challenges associated with traditional preservatives and the potential of natural alternatives to address these concerns. They provide an overview of the different types of essential oils and plant extracts that have been studied for their antimicrobial properties and review the literature on the use of these compounds to extend the shelf life of mushrooms.

Arense and Jideani [7] evaluated the effects of two different dipping solutions on the storage stability and consumer acceptability of dried apple slices for three months. One solution contained a mixture of citric acid) and *Moringa oleifera* leaf extract powder and the other solution contained critic acid, extract of moringa, and potassium sorbate. Tests were conducted on microbiology, acidity, moisture, water activity, texture, and colour. The solution effectively reduced browning and microbial growth, but moisture increased over time, which negatively affected the extensibility of the pre-treated dried sliced apples.

A study performed by Manged et al. [8] used Artificial Neural Networks (ANNs) to predict the pH, total soluble solids (TSS), water activity (aw), and moisture content (MC) of date fruits during cold storage based on their electrical properties. The ANN models were compared with Multiple Linear Regression (MLR) models at all testing frequencies of the electrical properties. The results showed that the ANNs models were more accurate in predicting fruit pH and had a better predictive ability for TSS, aw, and MC at all testing frequencies.

Liu et al. [9] pointed out that the transportation of apples can cause structural damage and deterioration due to extrusion and collision. The study considered the effects of extrusion distance, speed, working temperature, and apple variety on damage mechanisms The findings provide theoretical guidance for apple damage control during transportation.

Quintieri et al. [10] investigated the effectiveness of citral, a flavour component found in plant and fruit essential oils, against *Rhizopus oryzae* ITEM 18876. In vitro tests showed that citral was effective against this spoilage microorganism at concentrations equal to the minimal inhibitory concentration (MIC) and 4-fold MIC. Both concentrations were effective in preventing the microbial decay of infected table grapes over the storage period. The findings suggest that citral could be used to develop natural preservatives or active packaging to extend the shelf life of table grapes while preserving their quality and sensory characteristics, satisfying consumer demand for natural preservative agents.

Zongo et al. [11] explored the use of freeze-thawing (F-T) and pulsed electric field (PEF) pretreatments to modulate mango porosity and selectively enhance water removal over sugar gain during osmotic dehydration. Three different 60 °Brix agave syrup solutions, with or without added polysaccharides (inulin or xanthan gum), were used in the osmotic dehydration process. Results showed that PEF pretreatment slightly increased water loss (WL) during osmotic dehydration, while F-T led to a decrease. This study recommends using an osmotic solution with high viscosity to obtain low sugar uptake in the final product of F-T mangoes.

Muto et al. [12] studied six different cultivars of peach to assess changes in quality through nutritionally relevant compounds, aroma, physical characteristics, and sensorial evaluation. After 7 days of 1 °C storage, a trained panel evaluated the sensorial characters, while the carotenoids, phenolics, vitamin C, total sugars, and qualitative traits were integrated with volatile organic compound (VOC) analysis. This study found that the different analyses reveal interesting patterns of correlation, and the six cultivars responded differently to cold storage. The sensory parameters were correlated with 64 VOCs and seven intrinsic characters. The study also found that acidity, firmness, and 10 VOCs were negatively correlated with harmony and sweetness, but positively correlated with bitterness, astringency, and crunchiness. In contrast, Brix, b-carotene, and six VOCs were positively correlated with harmony and sweetness.

Liu et al. [13] investigated the effects of various LED lighting on the post-harvest firmness and nutritional quality of chili peppers. The research found that red and blue light could increase the content of capsaicinoids, while white and red light could increase the essential and aromatic amino acid content in pepper. The influence of light treatments on amino acid contents and compositions depended on the pepper genotype. Moreover, light affected fruit firmness and the content of nutrients such as chlorophyll, vitamin C, and total carotenoids, depending on the pepper genotypes. Thus, LED-light irradiation is a promising strategy for preserving or enhancing the post-harvest commercial and nutritional quality of pepper fruit.

Ren et al. [14] analysed the impact of selenium-chitosan on the quality of fresh-cut broccoli during storage. The results showed that selenium-chitosan treatment could slow down the reduction in Hue angle values and reduce ethylene release rate and respiration intensity. The molecular approach identified that this treatment could inhibit chlorophyll degradation and carotenoid biosynthesis, which would help to increase the shelf life of fruits and vegetables. This study provides insights into the molecular mechanism of selenium-chitosan in inhibiting the yellowing of fresh-cut broccoli.

Sun, et al. [15] studied the effects of different storage conditions, including temperature, light illumination, and low-temperature plasma treatments, on the water loss and quality of Gannan navel oranges were investigated. The results showed that storage at 5 °C could decrease the content of total soluble solids at the early storage stage and the content of titratable acids at the late storage stage, while storage at 26 °C could decrease the contents of total soluble solids at the late storage stage and the contents of titratable acids at the early storage stage. Additionally, low-temperature plasma treatment combined with storage at a low temperature under continuous red or blue light illumination was identified as a potential green technology to preserve Gannan navel oranges during storage.

Mo et al. [16] developed an eco-friendly method to improve the storability of harvested fruits using Magnolol loaded on carboxymethyl chitosan particles (Magnolol@CMCS) as a preservation coating agent. Magnolol@CMCS particles effectively solved the water insolubility and agglomeration issues of Magnolol and demonstrated greater toxicity against *Staphylococcus aureus*, *Escherichia coli*, and *Botryosphaeria dothidea* compared to Magnolol alone. Kiwifruit treated with Magnolol@CMCS showed delayed changes in fruit hardness and higher ascorbic acid and soluble total sugar contents. Magnolol@CMCS particles effectively improved the storability of kiwifruit.

Salas-Sanjuán et al. [17] evaluated the feasibility of hypobaric storage conditions for Marmande tomatoes. The results showed that sub-atmospheric storage led to a delay in ripening and a reduction in ethylene production. The degradation of chlorophylls was much slower in fruits stored at 75 kPa and 50 kPa compared to those stored at 101 kPa and control fruits. The sensory analysis also showed positive effects of sub-atmospheric treatments, suggesting that it could increase the shelf-life of fruits.

The various research mentioned above provides a comprehensive interdisciplinary perspective on the present status of postharvest preservation and fruit and vegetable quality. To create healthier, safer, and more sustainable horticultural products in the future, it will be imperative to focus on developing new technologies and innovative products.

## References

[1] Kumar P., Chatli M.K. (2018). Postharvest losses and strategies to reduce them: A review. J. Food Sci..

[2] Food and Agriculture Organization of the United Nations (2019). The State of Food and Agriculture. Moving forward on Food Loss and Waste Reduction.

[3] Ishangulyyev R., Kim S., Lee S.H. (2019). Understanding food loss and waste—Why are we losing and wasting food?. Foods.

[4] Bhardwaj S., Lata S., Garg R. (2022). Application of nanotechnology for preventing postharvest losses of agriproducts. J. Hort. Sci. Biotechnol..

[5] Chen S., Brahma S., Mackay J., Cao C., Aliakbarian B. (2020). The role of smart packaging system in food supply chain. J. Food Sci..

[6] Guo Y., Chen X., Gong P., Wang R., Han A., Deng Z., Qi Z., Long H., Wang J., Yao W. (2022). Advances in the role and mechanisms of essential oils and plant extracts as natural preservatives to extend the postharvest shelf life of edible mushrooms. Foods.

[7] Arendse W., Jideani V. (2022). Storage stability and consumer acceptability of dried apple: Impact of citric acid, potassium sorbate and moringa oleifera leaf extract powder. Foods.

[8] Manged M., Munir M., Aljabr A. (2022). Prediction of date fruit quality attributes during cold storage based on their electrical properties using artificial neural networks models. Foods.

[9] Liu X., Cao Z., Yang L., Chen H., Zhang Y. (2022). Research on damage properties of apples based on static compression combined with the finite Eeement method. Foods.

[10] Quintieri L., Fancello F., Caputo L., Sorrentino A., Zara S., Lippolis V., Cervelleri S., Fanelli F., Corvino A., Pace B. (2022). Effect of gaseous citral on table grapes contaminated by *Rhizopus oryzae* ITEM 18876. Foods.

[11] Zongo P.A., Khalloufi S., Mikhaylin S., Ratti C. (2022). Pulsed electric field and freeze-thawing pretreatments for sugar uptake modulation during osmotic dehydration of mango. Foods.

[12] Muto A., Christofides S.R., Sirangelo T.M., Bartella L., Muller C., Di Donna L., Muzzalupo I., Bruno L., Ferrante A., Chiappetta A. (2022). Fruitomics: The importance of combining sensory and chemical analyses in assessing cold storage responses of six peach (*Prunus persica* L. batsch) cultivars. Foods.

[13] Liu C., Wan H., Yang Y., Ye Q., Zhou G., Wang X., Ahammed G.J., Cheng Y. (2022). Post-Harvest LED Light irradiation affects firmness, bioactive substances, and amino acid compositions in chili pepper (*Capsicum annum* L.). Foods.

[14] Ren G., Liu Y., Deng B., Wang Y., Lin W., Zhang Y., Di J., Yang J. (2022). Gene expression analyses reveal mechanisms of inhibited yellowing by applying selenium-chitosan on fresh-cut broccoli. Foods.

[15] Sun Y., Li Y., Xu Y., Sang Y., Mei S., Xu C., Yu X., Pan T., Cheng C., Zang J. (2022). The effects of storage temperature, light illumination, and low-temperature plasma on fruit rot and change in quality of postharvest Gannan navel oranges. Foods.

[16] Mo F., Li W., Long Y., Li R., Ding Y., Li M. (2023). Magnolol loaded on carboxymethyl chitosan particles improved the antimicrobial resistance and storability of kiwifruits. Foods.

[17] Salas-Sanjuán M.C., Rebolloso M.M., Del Moral F., Valenzuela J.L. (2023). Use of sub-atmospheric pressure storage to improve the quality and shelf-life of Marmande tomatoes cv. Rojito. Foods.

